# The Effect of Anatomical Location of Lymph Node Metastases on Cancer Specific Survival in Patients with Clear Cell Renal Cell Carcinoma

**DOI:** 10.3389/fsurg.2018.00026

**Published:** 2018-03-28

**Authors:** Alessandro Nini, Alessandro Larcher, Francesco Cianflone, Francesco Trevisani, Carlo Terrone, Alessandro Volpe, Federica Regis, Alberto Briganti, Andrea Salonia, Francesco Montorsi, Roberto Bertini, Umberto Capitanio

**Affiliations:** ^1^Unit of Urology, University Vita-Salute San Raffaele, IRCCS San Raffaele Scientific Institute, Milan, Italy; ^2^Division of Oncology, Urological Research Institute (URI), Renal Cancer Unit, IRCCS San Raffaele Scientific Institute, Milan, Italy; ^3^Department of Urology, University Hospital Maggiore della Carità, University of Piemonte Orientale, Novara, Italy

**Keywords:** lymph node invasion, metastases, survival, kidney cancer, renal cancer

## Abstract

**Background:**

Positive nodal status (pN1) is an independent predictor of survival in renal cell carcinoma (RCC) patients. However, no study to date has tested whether the location of lymph node (LN) metastases does affect oncologic outcomes in a population submitted to radical nephrectomy (RN) and extended lymph node dissection (eLND).

**Objective:**

To describe nodal disease dissemination in clear cell RCC (ccRCC) patients and to assess the effect of the anatomical sites and the number of nodal areas affected on cancer specific mortality (CSM).

**Design, setting and partecipants:**

The study included 415 patients who underwent RN and eLND, defined as the removal of hilar, side-specific (pre/paraaortic or pre/paracaval) and interaortocaval LNs for ccRCC, at two institutions.

**Outcome measurement and statistical analysis:**

Descriptive statistics were used to depict nodal dissemination in pN1 patients, stratified according to nodal site and number of involved areas. Multivariable Cox regression analyses and Kaplan-Meier curves were used to explore the relationship between pN1 disease features and survival outcomes.

**Results and limitations:**

Median number of removed LN was 14 (IQR 9–19); 23% of patients were pN1. Among patients with one involved nodal site, 54 and 26% of patients were positive only in side-specific and interaortocaval station, respectively. The most frequent nodal site was the interaortocaval and side-specific one, for right and left ccRCC, respectively. Interaortocaval nodal positivity (HR 2.3, CI 95%: 1.3–3.9, *p* < 0.01) represented an independent predictor of CSM.

**Conclusions:**

When ccRCC patient harbour nodal disease, its spreading can occur at any nodal station without involving the others. The presence of interoartocaval positive nodes does affect oncologic outcomes.

**Patient summary:**

Lymph node invasion in patients with clear cell renal cell carcinoma is not following a fixed anatomical pattern. An extended lymph node dissection, during treatment for primary kidney tumour, would aid patient risk stratification and multimodality upfront treatment.

## Introduction

Positive nodal status and number of positive nodes are independent predictors of survival in renal cell carcinoma (RCC) patients (1, 2). Moreover, the role of lymph node dissection (LND) for RCC staging is widely accepted in intermediate/high risk patients, although its effect on cancer control is limited, especially in low risk patients (3). Adequate nodal staging and subsequent prognosis assessment become even more important in the light of the recent data published in the setting of adjuvant therapy, follow-up and salvage therapy (4–6).

The natural history of patients with nodal metastases is already known. Recently, it has been reported a 12% metastasis-free survival at 5 years (7). However, no study evaluated if prognosis is affected by location of the nodal metastasis or by the number of areas affected by nodal disease. This appears of paramount importance, if we consider that nodal invasion is often considered a criterion to define a RCC patient as metastatic, especially among medical oncologists.

Cadaveric dissection and sentinel-node studies (8–10) demonstrated wide heterogeneity of retroperitoneal lymphatic vessels anatomy. In addition, RCC histologies have different distant spreading rates (11) and oncologic outcomes (12, 13). Therefore, a critical analysis of nodal involvement areas in RCC patients submitted to RN and extended LND (eLND) might provide additional information on the pattern of lymphatic dissemination and its impact on the natural history of the disease.

Under such premises, the aim of this study was to describe nodal disease dissemination in clear cell RCC (ccRCC) patients and to assess the effect of the anatomical sites and the number of nodal areas affected by disease on cancer specific mortality (CSM).

## Materials and Methods

### Patient Population

**Figure 1:**
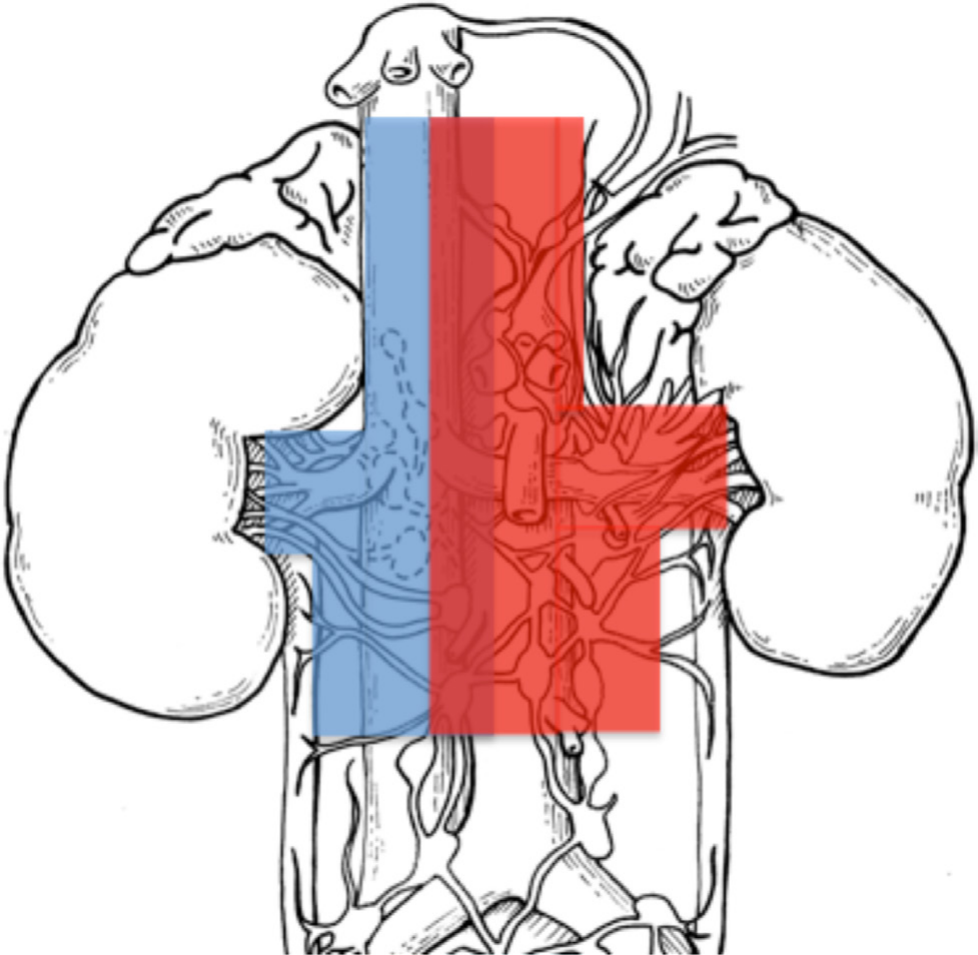
Sketch of left and right nodal templates (left template in red and right template in blue), adapted from (2).

After institutional ethic committee board approvals (IRCCS San Raffaele, Milan and Ospedale Maggiore della Carita´, Novara, Italy), we identified 2,884 patients with sporadic, unilateral, RCC treated with open radical nephrectomy (RN) between 1980 and 2012. Of these, 415 patients (14.4%) presented with clear cell RCC (ccRCC) histology and underwent RN plus eLND (San Raffaele 165/415, 40% and Novara 250/415, 60%), defined by a template including hilar, side-specific (pre/paraaortic or pre/paracaval) and interaortocaval nodal stations. All patients signed written informed consent to undergo surgery and to use clinical data in an anonymous fashion for scientific purposes. Nodal dissection template was shared between the Institutions, and ipsilateral template on the left side included nodes from the crus of the diaphragm to the inferior mesenteric artery, and on the right side, from the adrenal vein to the level of inferior mesenteric artery. Each nodal station was separately labelled and delivered to pathology (Figure 1).

The decision to perform LND was based on surgeon’s discretion and inclusion criteria were represented by cT2 disease and above, tumor size >10 cm, lymphadenopathies and palpable lymph nodes during surgery.

### Outcomes

Outcomes were other cause mortality (OCM)- and cancer specific mortality (CSM)-free survival rate. Secondly, a description of the pattern of nodal cancer dissemination in pN1 patient was performed on the overall ccRCC population and after stratification according to the kidney tumour side (right vs. left). Finally, we assessed the CSM-free survival rate according to the pattern of nodal dissemination (lymph node site or number of lymph node areas involved).

### Covariates

Covariates consisted of age, gender, tumour side (right vs. left), symptoms, clinical metastatic status (cM0 vs. cM1), pathological T stage (defined according to 7th American Joint Committee on Cancer classification) (14), pathological N stage, Fuhrman grade (according to the WHO/International Society of Urological Pathology classification) (15), tumour size, mean number of lymph nodes removed, number of positive lymph nodes, number of involved nodal sites, hilar, side-specific and interaortocaval nodal status.

### Statistical Analyses

Statistical analyses, as well as reporting and interpretation of the results, consisted of three steps.

Firstly, means, medians and interquartile ranges or frequencies and proportions were reported for continuous or categorical variables on the study population, respectively. Kaplan-Meier analyses were used to assess OCM- and CSM-free survival rate at different time points, on the overall population and after stratification for pN status.

Secondly, among patients with pN1 disease, a description of anatomical nodal involvement, stratified according to the number of the involved nodal sites (1 vs. 2 vs. 3) was performed.

Thirdly, among patients with pN1 disease, Cox multivariable regression analysis was used to predict the risk of CSM. Predictors consisted of number of involved lymph node areas, age, pT stage, pathologic tumour size, Fuhrman grade, and cM status. In an additional set of Cox multivariable regression analysis predicting CSM, anatomical nodal involvement (hilar vs. side-specific vs. interaortocaval nodal invasion) was used instead of number of involved lymph node areas. All statistical tests were performed using RStudio graphical interface v.0.98 for R software environment v.3.0.2 (R Foundation, Vienna, Austria). All tests were two-sided with a significance level set at p value < 0.05.

## Results

Clinicopathologic features are summarized in Table 1. The median number of removed lymph nodes (LNs) was 14 (IQR 9–19) and the median number of positive LNs was 3 (IQR 2–8). Overall, 74 patients (18%) and 179 patients (43%) had systemic and local symptoms, respectively. Clinical M1 status was present in 112 patients (27%), pT3/pT4 disease was found in 260 patients (63%), while 199 (48%) had Fuhrman grade 3/4.

**Table 1:** Descriptive statistics of 415 patients submitted to radical nephrectomy and extended LND with clear cell renal cell carcinoma.

**Variable**	**Overall**
**Mean age (years; median, IQR)**	57.6 (59, 55–66)
**Gender**	
Male	285 (68.7%)
Female	130 (31.3%)
**Symptoms**	
No symptoms	162 (39.0%)
Local symptoms	179 (43%)
Systemic symptoms	74 (18%)
**Site of primary tumour**	
Right	238 (57.3%)
Left	177 (42.7 %)
**Decade of surgery**	
1980–1989	108 (26%)
1990–1999	202 (48.7%)
2000–2009	80 (19.3%)
2010–2012	25 (6%)
**Metastatic status at diagnosis**	
cM0	303 (73%)
cM1	112 (27%)
**Pathologic T stage**	
T1a	26 (6.3%)
T1b	74 (17.8%)
T2a	43 (10.4%)
T2b	12 (2.9%)
T3a	100 (24.1%)
T3b	105 (25.3%)
T3c	28 (6.7%)
T4	27 (6.5%)
**Pathologic N stage**	
pN0	320 (77%)
pN1	95 (23%)
**Fuhrman grade**	
1	20 (4.8%)
2	174 (41.9%)
3	162 (39.0%)
4	37 (8.9%)
NA	22 (5.3%)
**Mean pathologic tumour size (cm; median, IQR)**	9 (8.5, 6–11.5)
**Mean number of nodes removed (median, IQR)**	15 (14, 9–19)
**Mean number of negative nodes (median, IQR)**	13 (12, 7–17)
**Mean number of positive nodes (median, IQR)**	5 (3, 2–8)
**Hilar nodal status**	
Negative	371 (89.4%)
Positive	44 (10.6%)
**Side-specific (paraaortic/pre- or paracaval) nodal status**	
Negative	342 (82.4%)
Positive	73 (17.6%)
**Interaortocaval nodal status**	
Negative	365 (88%)
Positive	50 (12%)
**Number of nodal sites involved**	
0	320 (77%)
1	46 (11.2%)
2	26 (6.3%)
3	23 (5.5%)
**Mean time to last follow-up or death (months; median, IQR)**	75.6 (43.7, 12.8–117)

**Figure 2:**
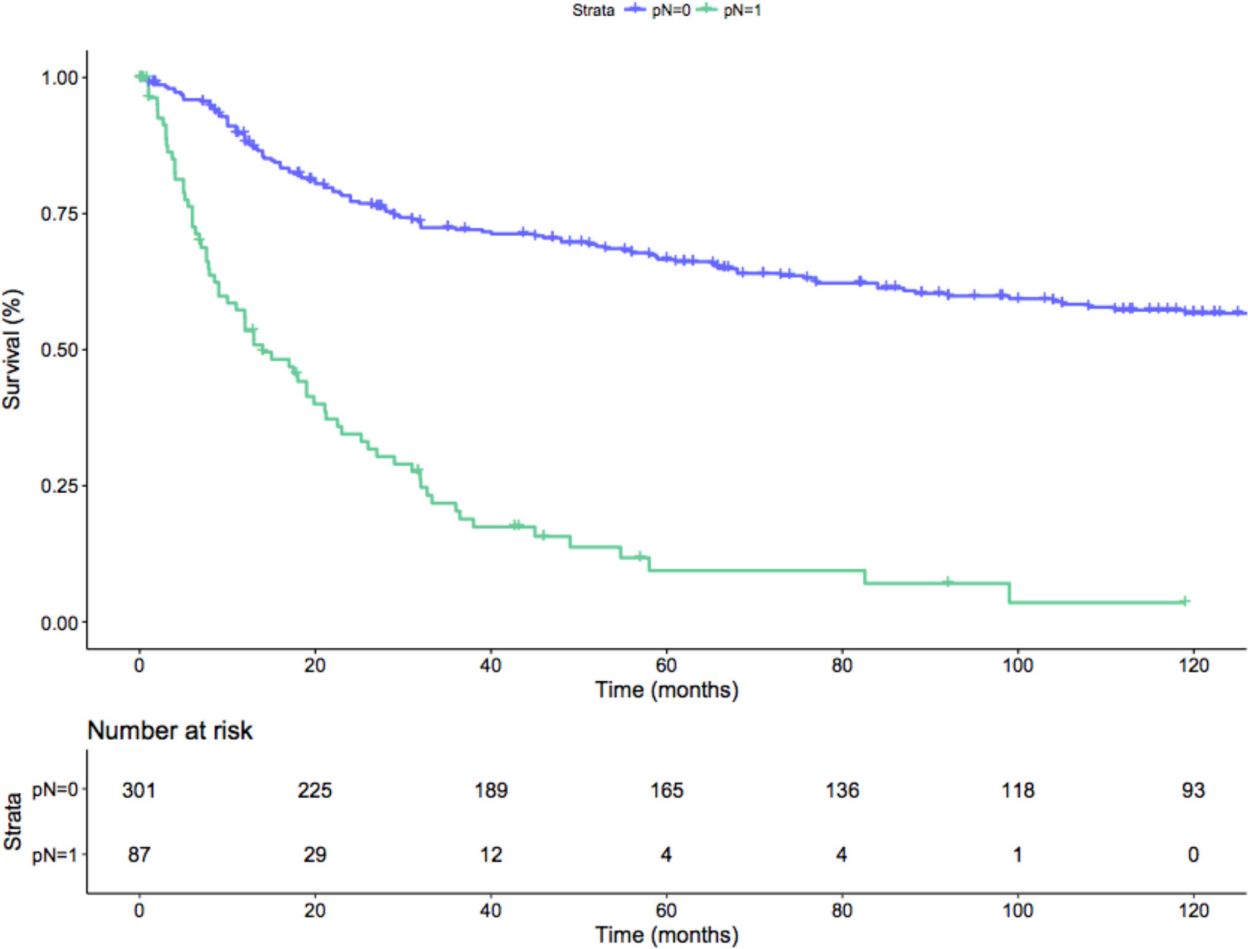
Kaplan-Meier depicting CSM-free survival rate on the overall population after stratification for the pN status (*p*-value < 0.01). Blue line: pN0 patients; Green line: pN1 patients.

Median follow-up among survivors was 43.7 months, with an overall OCM- and CSM-free survival rate of 91 and 60%. The 1 year, 3 year and 5 year OCM- and CSM-free survival rates were 97%, 93%, 90% and 80%, 61%, 55%, respectively. When patients were stratified according to nodal status (namely, pN0 and pN1), pN0 patients had 1 year, 3 year and 5 year CSM-free survival rates of 89, 72 and 66%, conversely pN1 patients’ CSM-free survival rates were 53, 20 and 9% (*p* < 0.01; Figure 2). Median time to CSM in pN0 cM0 patients was 30 months.

**Figure 3:**
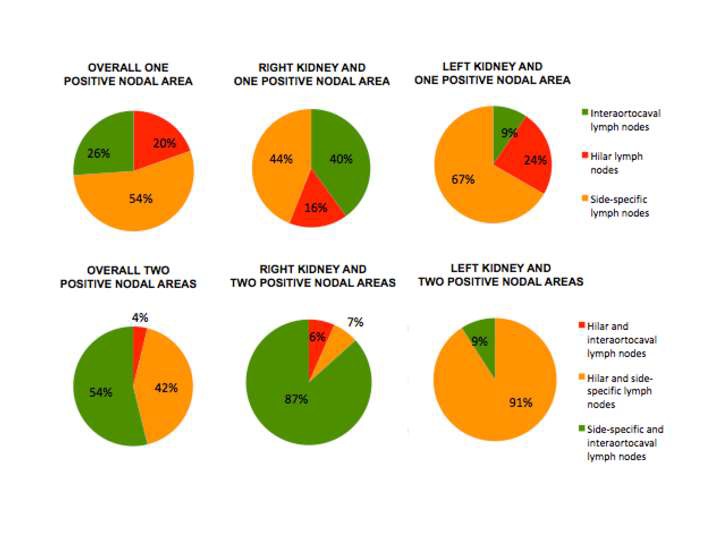
Nodal metastatic dissemination in the overall population and after stratification for the kidney site according to nodal areas and number of areas involved.

Locations of nodal metastases are summarized in Figure 3 and Table S1. Overall, within the group of patients with one positive nodal area, in 54% of patients metastatic dissemination skipped the hilar nodal area, while in 26% of cases, both the hilar and the side-specific areas were eluded. Instead, when looking at patients with two nodal areas involved, hilar nodal area was skipped in 54% of patients and side-specific area only in 4% of cases. Moreover after stratification for the ccRCC side, in case of interaortocaval-only nodal positivity among patients with one positive nodal site, 10 out of 12 patients had right ccRCC. This discrepancy was also identified in case of patients with two positive nodal areas and interaortocaval nodal involvement: the majority of patients had right ccRCC (14 out of 15 patients).

On Multivariable Cox regression analyses, when considering the location of nodal metastases, independent predictors of CSM were represented by pT3 (HR: 2.7; CI 95%: 1.5–5) and pT4 stage (HR: 6.1; CI 95%: 2.6–14.3), cM1 status (HR: 4.3; CI 95%: 3–6.2) and positive status of interaortocaval nodal area (HR: 2.3, CI 95%: 1.3–3.9), all *p* ≤ 0.01 (Table 2). Instead, when predicting CSM considering the number of positive nodal areas, independent predictors were pT3 (HR: 2.6; CI 95%: 1.4–4.8) and pT4 stage (HR: 4.2; CI 95%:1.9–9.6), pathologic tumour size (HR: 1.1; CI 95%: 1–1.1), cM1 status (HR: 4.2; CI 95%: 2.9–6) and presence of any number of nodal area involved (HR: 1.6–2.7; CI 95%: 1–5), all *p* < 0.05 (Table 3).

**Table 2:** Cox Logistic Regression analysis predicting CSM considering the location of nodal metastases.

	**UNIVARIABLE ANALYSES**		**MULTIVARIABLE ANALYSES**	
VARIABLES	HR (CI 95%)	*p*-value	HR (CI 95%)	*p*-value
Positive hilar nodes	4.1 (2.7–6)	<0.01	1.0 (0.6–1.8)	0.8
Positive side-specific nodes	4.2 (3–5.8)	<0.01	1 (0.6–1.6)	0.9
Positive interaortocaval nodes	5 (3.4–7.3)	<0.01	2.3 (1.3–3.9)	<0.01
Age	1 (0.9–1)	0.9	1 (0.9–1)	0.2
	1.8 (0.9–3.5)	0.09	1.2 (0.6–2.5)	0.6
pT3 vs. pT1	5.6 (3.4–9.3)	<0.01	2.7 (1.5–5)	0.01
pT4 vs pT1	21.6 (11.4–40.8)	<0.01	6.1 (2.6–14.3)	<0.01
Pathologic tumour size	1.1 (1.1–1.2)	<0.01	1 (1–1.1)	<0.01
Fuhrman grade	2.9 (2.1–4)	<0.01	1.2 (0.9–1.8)	0.2
Clinical metastatic status	7.5 (5.5–10.2)	<0.01	4.3 (3–6.2)	<0.01

**Table 3:** Cox Regression analysis predicting CSM considering the number of locations of nodal metastases.

	**UNIVARIABLE ANALYSES**		**MULTIVARIABLE ANALYSES**	
**VARIABLES**	**HR (CI 95%)**	*p*-value	**HR (CI 95%)**	*p*-value
Number of positive sites				
1 vs. 0	4.5 (3–6.7)	<0.01	1.6 (1–2.6)	<0.05
2 vs. 0	5.3 (3.3–8.7)	<0.01	1.7 (1–3)	<0.05
3 vs. 0	6.5 (3.7–11.4)	<0.01	2.7 (1.5–5)	<0.01
Age	1 (0.9–1)	0.9	1 (0.9–1)	0.2
pT stage				
	1.8 (0.9–3.5)	0.09	1.2 (0.5–2.4)	0.7
pT3 vs. pT1	5.6 (3.4–9.3)	<0.01	2.6 (1.4–4.8)	<0.01
pT4 vs. pT1	21.6 (11.4–40.8)	<0.01	4.2 (1.9–9.6)	<0.01
Pathologic tumour size	1.1 (1.1–1.2)	<0.01	1.1 (1–1.1)	<0.01
Fuhrman grade	2.9 (2.1–4)	<0.01	1.2 (0.9–1.8)	0.2
Clinical metastatic status	7.5 (5.5–10.2)	<0.01	4.2 (2.9–6)	<0.01

**Figure 4:**
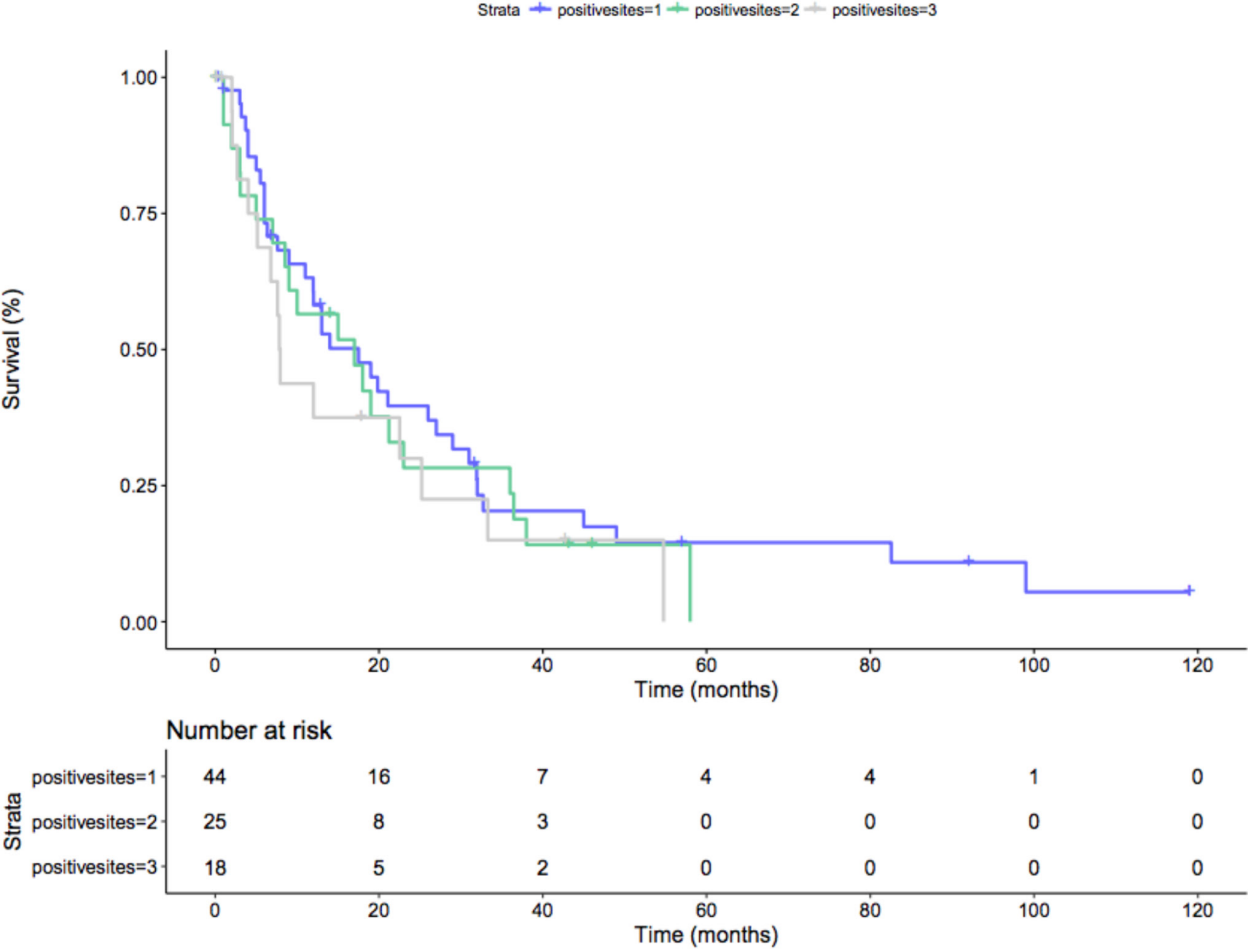
Kaplan-Meier depicting CSM-free survival rate only in pN+ patients after stratification for the number of locations of nodal metastases (*p* = 0.5). Blue line: 1 positive nodal site; Green line: 2 positive nodal sites; Grey line: 3 positive nodal sites.

However when considering only pN1 patients, no difference was seen at Kaplan-Meier analysis in terms of CSM-free survival rate, after stratification of number of involved nodal areas (*p* = 0.5; Figure 4).

## Discussion

Patients with nodal involvement in RCC have an 7.8-fold greater chance of CSM compared to pN0 counterparts (16, 17) and this has an independent prognostic value even in patient with metastatic RCC (1). Published retrospective studies (18) have indeed failed to reach an agreement on the topic. Moreover, the EORTC 30881 (19) did not demonstrate any benefit in terms of cancer control. Nevertheless, today, roughly 70% of that study population would have been classified as cT1abN0M0. In this regards, a subanalysis focusing only on cT3 tumours, showed a 15% overall survival benefit at 5 years for LND recipients (20). Therefore, EAU guidelines recognize the role of LND for cN1, although its extent remains controversial, and suggest an eLND for cN0 patients, only in presence of adverse clinical features (21). However, the picture appears even more complex, when considering that RCC histologies can differ in terms of distant spreading rates (11, 12) and oncologic outcomes (13).

Several observations of the current study are of importance. First, we described the oncologic outcomes of eLND in ccRCC patients. Cancer-specific mortality-free survival rates were worse for pN1 patients at any time point, compared to pN0 ones. According to Blute et al., among ccRCC patients, estimated CSM-free survival rates at 1-, 5- and 10 year follow-up were 95, 82 and 72.5% for pNx/pN0 patients and 52, 21 and 11% for pN +patients (16). Discrepancies with our results could be ascribed to inclusion of only cM0 ccRCC population and to omission of LND in some patients (42% of the overall population, data not shown for ccRCC histology). Moreover the study lacked of a definition for the extent of LND.

Second, for 54% of pN1 patients, disease eluded hilar nodal site (when considering patients with one or two positive nodal stations), in line with what has been previously reported by EAU guidelines (35–45%) (21). Only among patients with one positive nodal station, in 26% of patients, cancer skipped the hilar and the side-specific nodal stations. When looking at any patient with positive interaortocaval nodal station, the majority of them (*n* = 27/36, 75%) showed a right ccRCC. Focusing on those patients, with interaortocaval-only positive location of nodal metastases, 10/12 (83%) had right ccRCC. This observation is line with the studies investigating the role of sentinel LND (9, 10). In the first paper, 7/14 patients presented with interaortocaval nodal positivity: 3 of them had interoaortocaval and side-specific nodal positivity, 2 interaortocaval-only nodal positivity and 2 interaortocaval positivity and non-regional positivity; 6 of these patients had right kidney tumour. In the second, it was reported that right-sided tumour drained only to the paracaval nodes and left-sided tumours drained to the side-specific nodes.

It was formerly believed that RCC nodal drainage followed a fixed pattern, originating from the hilar region and branching off into the side-specific (paracaval/para-aortal) and the interaortocaval lymph nodes. Pioneering cadaveric and sentinel-node studies (8, 22–24) demonstrated extreme variations in drainage among RCC patients, even with aberrant firstly draining thoracic nodes. From the right kidney, efferent lymphatic vessels running anterior and posterior to the renal vein (anterior and posterior bundles) can drain into right side-specific and interaortocaval nodes. Retrocaval nodes connect with the thoracic duct but in some cases, efferent lymphatic vessels could reach the thoracic duct without passing through any lymph nodes. From the left kidney, efferent lymphatic vessels running anterior or posterior to the renal vein can drain into the left side-specific nodes. Posterior efferent lymphatic vessels could also connect directly to the thoracic duct without passing through any lymph node (8, 25, 26). These figures suggest that hilar LND could not be sufficient for every patient to be properly pN staged. Approximately, among patients with right ccRCC and single nodal positive site, 60% of them would have been properly staged as pN1 only by hilar and side-specific template, 40% only by eLND. Conversely, in left kidney tumours, 91% would have been properly staged as pN1 only by hilar and side-specific template, 9% only by eLND, in line with results from Crispen et al. (27).

Third, in an attempt to identify prognostic factors to aid patient risk stratification and multimodality upfront treatment, we found that cM1 disease, pT3-pT4 stage and positive interaortocaval nodal status and presence of any positive nodal area were independent predictors of CSM (all *p* ≤ 0.01). Taking into account the study on preoperative lymphoscintigraphy by Bex et al. (9), where 2/4 patients with interaortocaval-only nodal involvement had non-regional nodal involvement too, and the study by Brouwer et al. (22), where 1/4 patients with early lymphatic drainage of the thoracic duct had no retroperitoneal nodal metastasis, it is possible to state that eLND nodal dissection seems to be of utmost importance to properly stage patients and to tailor adjuvant treatment in the ccRCC population.

Despite several strengths, our analyses are not devoid of limitations. First, our report is intrinsically limited by their retrospective and noncomparative nature. Second, we could not match data to a control group of patients with ccRCC, who were not submitted to eLND. Third, over the years, many aspects, as for the administration and type of recommended adjuvant treatment, have been changing in the onco-surgical management of patients with ccRCC and nodal involvement. Four, central radiology and pathology revision of patient features was not possible. On the other hand, problems like analyses on any RCC histology and unstandardized LND template were overcome. Moreover, median number of resected lymph nodes was 14, which is considered the threshold to consider adequate an eLND. Specifically, by considering all locations of nodal metastases, the pattern of ccRCC metastatitic spread before reaching the thoracic duct has been drawn.

## Conclusion

At least, 40% of patients with right and 9% with left ccRCC would not been staged as pN1 by a LND limited to hilar and side-specific lymph nodes. Positive interaortocaval nodal status represented an independent predictor of CSM.

## Author Contributions

Conception and design of the work: UC, RB, CT, AV. Acquisition, analysis and interpretation of data: AN, AL, FR, FC, FT. Drafting the work: AN, AL, UC. Critical revision of the work for important intellectual content: UC, RB, CT, AV, FM, AS, AB.

## Conflict of Interest Statement

The authors declare that the research was conducted in the absence of any commercial or financial relationships that could be construed as a potential conflict of interest.
