# Supplementary material for: The Effect of Anatomical Location of Lymph Node Metastases on Cancer Specific Survival in Patients with Clear Cell Renal Cell Carcinoma
Source: Front Surg. 2018 Mar 28;5:26. doi: 10.3389/fsurg.2018.00026 (PMC5931172; doi:10.3389/fsurg.2018.00026)
Supplement: Supplementary file 1 [file DataSheet1.docx]

**Supplementary Table 1. Nodal metastatic dissemination in the overall population and after stratification for the kidney site according to nodal areas and number of areas involved**

| **OVERALL POPULATION (n=95)** | **Number (%)** |
| --- | --- |
| **One positive nodal site** | 46 (100%) |
| Only hilar lymph nodes | 9 (20%) |
| Only side specific lymph nodes | 25 (54%) |
| Only interaortocaval lymph nodes | 12 (26%) |
| **Two positive nodal sites** | 26 (100%) |
| Hilar and interaortocaval lymph nodes | 1 (4%) |
| Hilar and side specific lymph nodes | 11 (42%) |
| Interaortocaval and side specific lymph nodes | 14 (54%) |
| **Three positive nodal sites** | 23 (100%) |
| Hilar and interaortocaval and side specific lymph nodes | 23 (100%) |
| **RIGHT KIDNEY TUMOUR (n=56)** | **Number (%)** |
| **One positive nodal site** | 25 (100%) |
| Only hilar lymph nodes | 4 (16%) |
| Only side specific lymph nodes | 11 (44%) |
| Only interaortocaval lymph nodes | 10 (40%) |
| **Two positive nodal sites** | 15 (100%) |
| Hilar and interaortocaval lymph nodes | 1 (6.5%) |
| Hilar and side specific lymph nodes | 1 (6.5%) |
| Interaortocaval and side specific lymph nodes | 13 (87%) |
| **Three positive nodal sites** | 16 (100%) |
| Hilar and interaortocaval and side specific lymph nodes | 16 (100%) |
| **LEFT KIDNEY TUMOUR (n=39)** | **Number (%)** |
| **One positive nodal site** | 21 (100%) |
| Only hilar lymph nodes | 5 (24%) |
| Only side specific lymph nodes | 14 (67%) |
| Only interaortocaval lymph nodes | 2 (9%) |
| **Two positive nodal sites** | 11 (100%) |
| Hilar and interaortocaval lymph nodes | 0 (0%) |
| Hilar and side specific lymph nodes | 10 (90%) |
| Interaortocaval and side specific lymph nodes | 1 (10%) |
| **Three positive nodal sites** | 7 (100%) |
| Hilar and interaortocaval and side specific lymph nodes | 7 (100%) |
